# Temporal patterns of inflammatory gene expression in local tissues after banding or burdizzo castration in cattle

**DOI:** 10.1186/1746-6148-5-36

**Published:** 2009-09-23

**Authors:** Wanyong Pang, Bernadette Earley, Torres Sweeney, Vivian Gath, Mark A Crowe

**Affiliations:** 1 Teagasc, Animal Bioscience Research Centre, Dunsany, Co. Meath, Ireland; 2 School of Agriculture, Food Science & Veterinary Medicine, University College Dublin, Belfield, Dublin 4, Ireland

## Abstract

**Background:**

Castration of male cattle has been shown to elicit inflammatory reactions and acute inflammation is initiated and sustained by the participation of cytokines.

**Methods:**

Sixty continental × beef bulls (Mean age 12 ± (s.e.) 0.2 months; Mean weight 341 ± (s.e.) 3.0 kg) were blocked by weight and randomly assigned to one of three treatments (n = 20 animals per treatment): 1) untreated control (Con); 2) banding castration at 0 min (Band); 3) Burdizzo castration at 0 min (Burd). Samples of the testis, epididymis and scrotal skin were collected surgically from 5 animals from each group at 12 h, 24 h, 7 d, and 14 d post-treatment, and analysed using real-time PCR. A repeated measurement analysis (Proc GLM) was performed using SAS. If there was no treatment and time interaction, main effects of treatment by time were tested by ANOVA.

**Results:**

Electrophoresis data showed that by 7 d post-castration RNA isolated from all the testicle samples of the Burd castrated animals, the epididymis and middle scrotum samples from Band castrates were degraded. Transitory effects were observed in the gene expression of IFN-γ, IL-6, IL-8 and TNF-α at 12 h and 24 h post treatment. Burd castrates had greater (P < 0.05) testicular IFN-γ mRNA levels compared with Band and Con animals, but lower (P < 0.05) testicular TNF-α mRNA levels compared with Con animals. Band castrates had greater (P < 0.05) testicular IL-6 mRNA levels than Burd castrates at 12 h post-castration. Burd castrates had greater (P < 0.05) testicular IL-8 mRNA levels than Band and Con animals at 24 h post-castration. In the epididymis, Burd castrates had greater (P < 0.05) IL-6 mRNA (both at 12 h and 24 h post treatment) and IL-8 mRNA (12 h post treatment) levels compared with Band and Con animals; Burd castrates had greater (P = 0.049) IL-10 mRNA levels than Band castrates at 12 h post-castration.

**Conclusion:**

Banding castration caused more inflammatory associated gene expression changes to the epididymis and scrotum than burdizzo. Burdizzo caused more severe acute inflammatory responses, in terms of pro-inflammatory cytokine gene expression, in the testis and epididymis than banding.

## Background

Techniques used to castrate male cattle include the application of rubber rings or tightened latex bands (referred to as banding) [1,2], surgical removal of the testicles [3] and use of a burdizzo instrument to crush the testicular cords [4]. Castration of male cattle has been shown to elicit physiological stress, inflammatory reactions, pain-associated behaviour, suppression of immune function, and a reduction in performance [5-7].

Banding or burdizzo castration causes ischemia, which in turn leads to necrosis and inflammation. Acute inflammation is initiated and sustained by the participation of cytokines including tumor necrosis factor α (TNF-α), interleukin-1 (IL-1), 6, and interferon-gamma (IFN-γ) [8]. In later stages of inflammation, cytokines (e.g., IL-10) are produced by infiltrating leukocytes that limit inflammation and counteract hyperalgesia [9]. IL-8 was identified as an agonist for neutrophils [10] and neutrophils are involved in many stress models [11-14]. It was reported that acute phase cytokine expression including IL-1β, IL-1Ra, and TNF-a can be altered in dairy calves treated with dexamethasone, which may decrease resistance of those animals to disease [15].

There is currently no data available in the literature on the effects of banding or burdizzo castration on inflammatory gene expression, which would be an indicator of the severity of inflammation, in the local tissue around the castration site. To study the inflammatory gene profiles would help monitor the local inflammation resulting from castration procedures and aid in scheduling possible post-castration care to alleviate animal suffering. The hypothesis was that bovine local tissues around the castration site may be responding to the changed blood flow (ischemia) associated with banding or burdizzo castration by altering the expression of cytokine genes involved in inflammatory functions. The objective was to monitor local tissue inflammatory cytokine gene expression profiles (IL-1, 6, 8, 10, IFN-γ, TNF-α) using quantitative real-time RT-PCR.

## Results

### RNA stability post-castration

**Figure 1:**
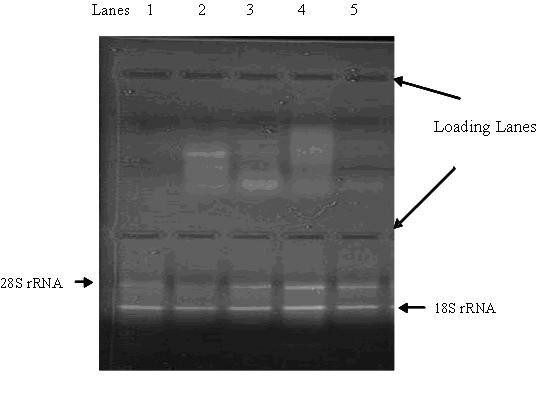
**Electrophoresis of middle scrotum samples (168 h post castration)**. Upper lanes: 5 samples of Band castrates (degraded 28S and 18S bands). Lower lanes: 5 samples of Burd castrates (intact 28S and 18S bands).

The 28 S and 18 S rRNA bands were degraded in all tissues of all band-castrated animals and in the testicular samples of burdizzo castrated animals by day 7 (Figure 1). At 14 d post-castration, all the RNA samples from both band and burdizzo-castrated animals were degraded. RNA degraded tissues were excluded from real-time PCR assays. All other RNA samples had spectrophotometer absorbance ratios (260:280) in the range of 1.8 to 2.3.

### Gene expression in the band castrated animals (Figures 2 and 3)

**Figure 2:**
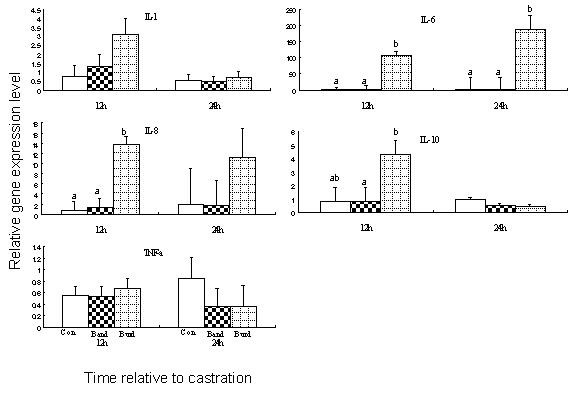
**Mean relevant level (± SEM) of cytokine genes in the epididymis for bulls left untreated (Con), or castrated by banding (Band) or burdizzo (Burd)**. 12 h post castration, Burd animals had greater (P < 0.05) IL-6 mRNA level than Band and Con animals; Burd animals had greater (P < 0.05) IL-8 mRNA level than Band and Con animals; Burd animals had greater (P < 0.05) IL-10 mRNA level than Band animals. 24 h post castration, Burd animals had greater (P < 0.05) IL-6 mRNA level than Band and Con animals; ^a, b^Means within a row (by time) that do not have common superscripts differ (P < 0.05).

**Figure 3:**
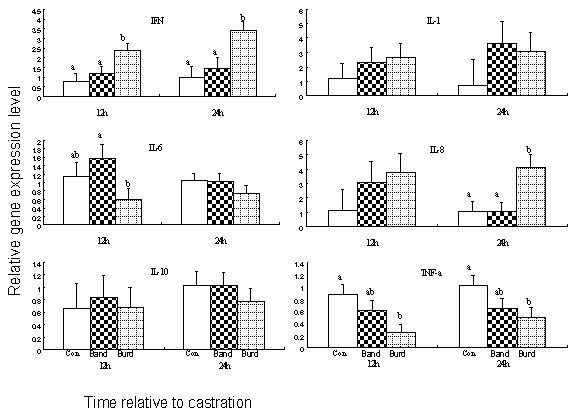
**Mean relevant level (± SEM) of cytokine genes in the testis for bulls left untreated (Con), or castrated by banding (Band) or burdizzo (Burd)**. 12 h post castration, Burd animals had greater (P < 0.05) IFN-γ mRNA level than Band and Con animals; Band animals had greater (P < 0.05) IL-6 mRNA level than Burd animals; Burd animals had lower (P < 0.05) TNF-α mRNA level than Con animals. 24 h post castration, Burd animals had greater (P < 0.05) IFN-γ mRNA level than Band and Con animals; Burd animals had greater (P < 0.05) IL-8 mRNA level than Band and Con animals; Burd animals had lower (P < 0.05) TNF-α mRNA level than Con animals. ^a, b^Means within a row (by time) that do not have common superscripts differ (P < 0.05).

There was no evidence for altered mRNA expression of the pro- or anti-inflammatory cytokines in any of the tissues examined (testes, epididymis and skin) of band castrated animals at either 12 or 24 hours post-castration.

### Gene expression in the burdizzo castrated animals (Figures 2 and 3)

Burdizzo castration was associated with the increased mRNA expression of the pro-inflammatory cytokines IL-6 (12 h, 24 h and 168 h), and IL-8 and the anti-inflammatory cytokine IL-10 (12 h) in the epididymis, while IFN-γ gene expression was not detected. Burdizzo castration was associated with the increased expression of the pro-inflammatory cytokines INF-γ (12 and 24 h) and IL-8 (24 h) in the testes, while there was a reduction in the expression of TNF-α at both 12 and 24 h. Expression levels for the genes evaluated in the scrotal skin (ST and SM) were similar in burdizzo castrates at all time points to that of controls. TNF-α gene expression was not influenced in the epididymal tissue by burdizzo castration.

## Discussion

The cytokine gene expression profiles in scrotal tissues associated with acute trauma post-castration were examined in this study. Interestingly, there was no evidence for altered inflammatory cytokine gene expression in the tissues examined in band-castrated animals. In contrast, a variety of pro- and anti-inflammatory cytokines were elevated in the epididymal tissues of burdizzo-castrated animals. The mRNA expression profiles of the cytokines analysed in the epididymis of burdizzo-castrated animals was consistent with what has been previously been described in a tissue trauma cytokine profile [8].

There was no difference in TNF-α gene expression levels in the epididymis at all time points examined in this study. TNF-α is one of the first cytokines released following tissue injury and has an array of biological activities including increasing vascular permeability, stimulation of acute phase protein secretion and induction of a spectrum of pro-/and anti-inflammatory cytokines such as IL-6, IL-8, INF-γ, and IL-10. While TNF-α is produced by activated monocytes and macrophages, its expression has also been identified in pachytene spermatocytes and round spermatids [16], and in activated macrophages isolated from the testis [17-19]. Our observation that TNF-α was not expressed at 12 h, 24 h or 168 h is consistent with the concept that TNF-α is one of the earliest cytokines activated in the tissue injury response. Studies on experimental traumatic brain injury models have demonstrated robust increases in TNF-α at the injured sites within a few hours post injury, and this cytokine had returned to baseline by 24 h [20-22]. TNF-α had most probably been upregulated at an earlier time point and had induced an array of pro and anti-inflammatory cytokines, but, by 12 h is now itself down regulated under the influence of elevated levels of anti-inflammatory agents such as IL-6, which was elevated at 12 h in the epididymal tissue. TNF-α acts synergistically with another early response pro-inflammatory cytokine, IL-1. These two cytokines act via a common signalling pathway [23] leading to increased permeability and coagulation ability of the endothelium as well as the induction of a spectrum of inflammatory and immune response genes. Following this pattern, IL-1 was upregulated at 12 h, but down regulated in the 24 h and 168 h tissue samples.

IL-6 is a complex regulatory cytokine. It is initially a pro-inflammatory cytokine, but after a period of elevated concentrations, it stimulates an anti-inflammatory process through its regulatory influence on the TNF-α and IL-1 receptors. Our results are in keeping with this role as we observed continuously elevated levels of IL-6 in the epididymal tissues of burdizzo castrated animals at all time points examined. Placing our data in the context of the described role in the literature, this suggests that IL-6 is acting as a potent anti-inflammatory throughout the latter stages of burdizzo castration. The combined pattern of gene expression of IL-1, IL-6 and TNF-α is of interest to stress models such as castration as several lines of evidence suggest that the acute phase cytokines IL-1, IL-6 and TNF-α may act synergistically in the neuroendocrine system, and influence the release of adrenocorticotropic hormone (ACTH) and corticosterone by potentiating each other's actions [24,25].

IL-10 is a recognised anti-inflammatory cytokine [8]. It participates in the down regulation of inflammatory immune responses by hampering activation of macrophages and dendritic cells and thus inhibiting TH1 responses. IL-10 is also an inhibitor of IFN-γ. Interestingly, IL-10 has traditionally been shown to be produced by activated macrophages [26]. IL-10 was up regulated at 12 h in Burd castrated animals. Because most macrophages are settled within tissues the increased abundance of IL-10 gene observed in Burd castrated animals might have derived from T lymphocytes, which are known to produce IL-10 [26]. Overall, the dramatic up regulation of IL-10 in Burd castrated animals (at 12 h post-castration) may indicate an attempt by the animal's immune system to reduce inflammation. Thus, during inflammation, analgesic cytokines counteract the effects of the proinflammatory hyperanalgesic cytokines generated in the early stages of the inflammatory response. In addition to the importance of inflammation as a means of immune defense and healing, strong inhibitory mechanisms to the inflammatory process are needed to avoid uncontrolled inflammation and consequent severe tissue damage.

IL-8 is a chemotactic cytokine that is produced at an early stage of inflammation with the aim of recruiting neutrophils to the trauma site. Its expression can persist for weeks. There was a greater level of IL-8 mRNA expression in both the testes and epididymis of Burdizzo castrated than Band castrated animals in this experiment. This suggests that burdizzo castration led to a greater acute inflammatory response than banding, because IL-8 enhances inflammation by enabling immune cells to migrate into tissues and is a powerful inducer of chemotaxis for neutrophils.

Another interesting aspect of the study is that TNF-α gene expression levels were lower in Burd castrated animals than controls at 12 h and 168 h post-castration. We propose that the cytokines released/presented at the tissue sites in burdizzo castrated animals might play important roles in mobilizing peripheral inflammatory cells into damaged tissue. Further studies will be required to fully explore the complex roles of neutrophil accumulation with increased levels of TNF-α after an acute inflammatory response

The early degradation of the 28S and 18S rRNA in band castrated animals, suggests that banding castration induced a more rapid loss of cell function. This suggests that band castration caused an earlier necrosis or atrophy damage to the epididymis and scrotum than burdizzo castration. This is in accordance with the different principles of these two castration techniques. Banding causes ischemia to testicles, epididymis and also scrotum, leading to ischemic necrosis of the testicles, and eventually testicular atrophy and sloughing of the scrotum. Burdizzo castration is based on the principle that crushing destroys the spermatic cord carrying blood to the testicles but that the skin of the scrotum remains intact, as clamping of the burdizzo on each spermatic cord is not overlapped [1,4].

Studies on experimental traumatic brain injury models have demonstrated robust increases in proinflammatory cytokine TNF-α at the injured sites within a few hours post-injury, and this cytokine returned to baseline by 24 h [20-22].

Another interesting finding in the study is that TNF-α gene expression levels were lower in Burd castrated animals than control at 12 h and 168 h post-castration. We propose that the cytokines released/presented at the tissue sites in burdizzo castrated animals might play important roles in mobilizing peripheral inflammatory cells into damaged tissue. Further studies will be required to fully explore the complex roles of neutrophil accumulation with increased levels of TNF-α after an acute inflammatory response.

## Conclusion

In conclusion, banding castration induced a more rapid loss of cell function (as evidenced by the earlier degradation of rRNA) compared with burdizzo castration. Burdizzo castration caused a more severe acute inflammatory response than banding castration in the testis and epididymis, in terms of pro-inflammatory cytokine gene expression. Secondary injury mechanisms, including inflammation, are important therapeutic targets for protection of tissues, and understanding the mediators of inflammation is important as new therapeutic strategies are developed for pain relief during castration. Importantly, protein levels as well as other biomarkers require future consideration when investigating the underlying mechanisms of tissue trauma post-castration.

## Methods

### Treatments

Sixty continental × beef bulls (Mean age 12 ± (s.e.) 0.2 months; Mean weight 341 ± (s.e.) 3.0 kg) were blocked by weight, and randomly assigned to one of three treatments (20 animals/treatment): 1) untreated control (Con); 2) banding castration at 0 min (Band); 3) Burdizzo castration at 0 min (Burd).

### Animal Housing and Management

Bulls were housed in individual tie-stalls from d -14 (day of treatment = d 0) to acclimatize them to handling, restraint and their housing environment. Animals had *ad libitum *access to water and grass silage.

### Experimental Procedures

Band animals were castrated (time = 0 min) with latex rubber bands applied and tensioned to the neck of the scrotum using the Callicrate Smart Bander (St. Francis, KS, USA) following the instrument guidelines. Burdizzo castration (time = 0 min) was performed in the Burd animals [6] with 10 sec crushing of the burdizzo applied to each spermatic cord. As part of the castration procedure, gentle manual restraint of the bulls was used to facilitate the operator. Animals in the control group were sham handled for a period equivalent to the time required to perform the castration procedure in the treatment groups.

**Figure 4:**
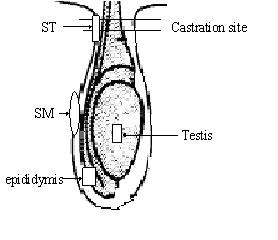
**Diagrammatic representation of tissue sampling sites**. ST: skin around the procedure site; SM: the middle scrotum.

At the time of tissue sampling, a latex rubber band was applied above the Band or Burd castration site or equivalent site in control animals, so as to avoid ascending infection from the castration site. At each time point of 12 h, 24 h, 7 d (168 h), and 14 d post-treatment, samples of the testis, epididymis and scrotal skin (skin around the procedure site (ST), and the middle scrotum (SM)) were collected surgically from 5 animals from each group (Figure 4). Tissue samples were dissected and frozen in liquid nitrogen immediately and then stored at -80°C until analyzed. All procedures were conducted under experimental license from the Irish Department of Health in accordance with the Cruelty to Animals Act 1876, and the European Communities (Amendment of Cruelty to Animals Act 1876) Regulations, 1994.

### Assay procedures

Tissue samples were broken into small pieces after removal from the liquid nitrogen. After weighing, tissue samples (~50 mg) were placed in a liquid nitrogen pre-cooled 14 mL round-bottom culture tube, followed by addition of 1 mL TRIzol Reagent (Invitrogen, Carlsbad, CA, USA). Tissues were homogenized using a Polytron homogenizer (KINEMATICA AG, Switzerland). Total RNA was isolated from the tissue suspension following TRIzol Reagent manufacturer's instructions. The isolated RNA was DNase (Promega, WI, USA) treated, re-extracted using phenol/chloroform (Sigma-Aldrich, MO, USA), followed by precipitation using RNase-free 3 M sodium acetate and pure ethanol (Sigma-Aldrich, Dublin, Ireland). Following isolation and purification, RNA concentration and purity were determined using an UV-1601 PC spectrophotometer (SHIMADZU, Japan) set at 260 and 280 nm readings. RNA quality was also checked by 28S and 18S rRNA band visualization following gel electrophoresis. RNA was converted into first-strand cDNA using SuperScript™ III First-Strand Synthesis System (Invitrogen) following the manufacturer's instructions.

Quantitative real-time PCR was performed using the SYBR Green PCR master Mix (Applied Biosystems, UK). Seven gene-specific primer pairs (Table 1) were designed using Primer Express Software (Applied Biosystems) and synthesized at MWG (UK). β-actin was included as an endogenous reference in all real-time PCR analyses. The real-time PCR was performed using an automated fluorometer (ABI Prism 7700 Sequence Detection System, Applied Biosystems). A 10 μL sample containing 0.5 μL cDNA, 300 nM of forward and reverse primers, and SYBR Green PCR master Mix, was placed in each well of a 384-well plate. The samples were run in triplicate. Real-time PCR conditions were as follows: stage 1 of 50°C for 2 min; stage 2 of 95°C for 10 min and followed by stage 3 of 40 repeats of 95°C for 15 sec and 60°C for 1 min. Results were recorded as relative gene expression changes after normalising for β-actin gene expression, and calculated using the 2^-ΔΔCt ^method [27]. The average C_T _values of control animals at each time point were the calibrator used to determine relative gene expression changes.

**Table 1:** Sequences (5'-3') of forward and reverse primers used in real-time PCR

**Gene**	**Forward primer sequence**	**Reverse primer sequence**
IL-1α	TTGGTGCACATGGCAAGTG	GCACAGTCAAGGCTATTTTTCCA
IL-6	GCGCATGGTCGACAAAATCT	TGTCTCCTTGCTGCTTTCACA
IL-8	TGGGCCACACTGTGAAAATTC	CCTTCTGCACCCACTTTTCC
IL-10	CTTGTCGGAAATGATCCAGTTTT	TCAGGCCCGTGGTTCTCA
IFN-γ	TGGAGGACTTCAAAAAGCTGATT	TTTATGGCTTTGCGCTGGAT
TNF-α	TCTACCAGGGAGGAGTCTTCCA	GTCCGGCAGGTTGATCTCA
β-actin	CGCCATGGATGATGATATTGC	AAGCGGCCTTGCACAT

### Statistical analyses

All statistical analyses were performed using SAS V 9.1 (SAS Institute Inc. NC, USA). A repeated measurement analysis (Proc GLM) was performed. If there was no treatment and time interaction, main effects of treatment × time were tested by ANOVA. A probability of *P *< 0.05 was chosen as the level of significance for the statistical tests.

## Authors' contributions

WP carried out the animal studies, RNA extraction, real-time PCR, performed the statistical analysis, and drafted the manuscript. BE and MC conceived the study, participated in its design and coordination and contributed to review and writing the manuscript. VG and MC carried out the castrations and tissue sampling. TS designed and helped with real-time PCR analysis. All authors read and approved the final manuscript.

## References

[B1] Fell LR, Wells R, Shutt DA (1986). Stress in calves castrated surgically or by the application of rubber rings. Aust Vet J.

[B2] Chase CC, Larsen RE, Randel RD, Hammond AC, Adams EL (1995). Plasma cortisol and white blood cell responses in different breeds of bulls: A comparison of two methods of castration. J Anim Sci.

[B3] Jennings PB (1984). Testicular surgery. The Practice of Large Animal Surgery.

[B4] Robertson IS, Kent JE, Molony V (1994). Effect of different methods of castration on behaviour and plasma cortisol in calves of three ages. Res Vet Sci.

[B5] Molony V, Kent JE, Robertson IS (1995). Assessment of acute and chronic pain after different methods of castration of calves. Appl Anim Behav Sci.

[B6] Fisher AD, Crowe MA, Alonso de la Varga ME, Enright WJ (1996). Effect of castration method and the provision of local anaesthesia on plasma cortisol, scrotal circumference, growth and feed intake of bull calves. J Anim Sci.

[B7] Pang WY, Earley B, Sweeney T, Crowe MA (2006). Effect of carprofen administration during banding or burdizzo castration of bulls on plasma cortisol, in vitro interferon-γ production, acute-phase proteins, feed intake, and growth. J Anim Sci.

[B8] Cheville NF (1999). Introduction to Veterinary Pathology.

[B9] Cunha FQ, Ferreira SH (2003). Peripheral hyperalgesic cytokines. Adv Exp Med Biol.

[B10] Baggiolini M, Dewald B, Moser B, Gallin JI, Snyderman R (1999). Chemokines. Inflammtion Basic principles and clinical correlates.

[B11] Murata H (1997). Effects of burdizzo castration on peripheral blood lymphocyte parameters in calves. Vet J.

[B12] Barton AE, Bayley DL, Mikami M, Llewellyn-Jones CG, Stockley RA (2000). Phenotypic changes in neutrophils related to anti-inflammatory therapy. Biochim Biophys Acta.

[B13] Weber PSD, Madsen SA, Smith GW, Ireland JJ, Burton JL (2001). Pre-translational regulation of neutrophil L-selectin in glucocrticoid-challenged cattle. Vet Immunol Immunopathol.

[B14] Ting STL, Earley B, Crowe MA (2004). Effect of cortisol infusion patterns and castration on metabolic and immunological indices of stress response in cattle. Dom Anim Endocrinol.

[B15] Eicher SD, McMunn KA, Hammon HM, Donkin SS (2004). Toll-like receptors 2 and 4, and acute phase cytokine gene expression in dexamethasone and growth hormone treated dairy calves. Vet Immunol Immunopathol.

[B16] De SK, Chen HL, Pace JL, Hunt JS, Terranova PF, Enders GC (1993). Expression of tumor necrosis factor-α in mouse spermatogenic cells. Endocrinol.

[B17] Xiong Y, Hales DB (1993). Expression, regulation, and production of tumor necrosis factor-a in mouse testicular interstitial macrophages in vitro. Endocrinol.

[B18] Xiong Y, Hales DB (1994). Immune-endocrine interactions in the mouse testis: cytokinemediated inhibition of Leydig cell steroidogenesis. Endocr J.

[B19] Moore C, Hutson JC (1994). Physiological relevance of tumor necrosis factor in mediating macrophage-Leydig cell interactions. Endocrinol.

[B20] Fan L, Young PR, Barone FC, Feuerstein GZ, Smith DH, McIntosh TK (1996). Experimental brain injury induces differential expression of tumor necrosis factor-alpha mRNA in the CNS. Brain Res Mol Brain Res.

[B21] Knoblach SM, Fan L, Faden AI (1999). Early neuronal expression of tumor necrosis factor-alpha after experimental brain injury contributes to neurological impairment. J Neuroimmunol.

[B22] Vitarbo EA, Chatzipanteli K, Kinoshita K, Truettner JS, Alonso OF, Dietrich WD (2004). Tumor necrosis factor alpha expression and protein levels after fluid percussion injury in rats: The effect of injury severity and brain temperature. Neurosurgery.

[B23] Tsirogianni AK, Moutsopoulos NM, Moutsopoulos HM (2006). Wound healing: Immunological aspects. Injury.

[B24] Buckingham JC (1996). Fifteenth Gaddum Memorial Lecture December 1994. Stress and the neuroendocrine-immune axis: the pivotal role of glucocorticoids and lipocortin 1. Br J Pharmacol.

[B25] Turnbull AV, Rivier CL (1999). Regulation of the hypothalamic-pituitary-adrenal axis by cytokines: actions and mechanisms of action. Physiol Rev.

[B26] Abbas AK, Lichtman AH (2003). Cellular and molecular immunology.

[B27] Livak KJ, Schmittgen TD (2001). Analysis of relative gene expression data using real-time quantitative PCR and the 2(-Delta Delta C(T)) Method. Methods.

